# AI-Enabled Modeling for Alzheimer’s Disease Risk Prediction and Validation

**DOI:** 10.31083/RN49220

**Published:** 2026-07-21

**Authors:** Pingping Li, Yida Wang

**Affiliations:** ^1^School of Medicine, Xuchang University, 461000 Xuchang, Henan, China; ^2^School of Public Health, Shandong Medical and Pharmaceutical University, 264003 Yantai, Shandong, China

**Keywords:** Alzheimer’s disease, machine learning, risk prediction model, random forest

## Abstract

**Background:**

To investigate the multimodal clinical influencing factors of Alzheimer’s disease (AD) onset, and to establish and test a risk prediction tool derived from these determinants and additional clinical measures, thus facilitating early intervention and risk classification in individuals at high risk for AD.

**Methods:**

A retrospective cohort of 502 high-risk individuals for AD (exhibiting cognitive decline or family history) who visited our hospital was included. A total of 502 participants were randomly split into a training cohort (n = 350) and a validation cohort (n = 152) in a 7:3 proportion. Demographic characteristics, clinical indicators, biomarkers, and genetic markers were collected. In the training set, univariate analysis and least absolute shrinkage and selection operator (LASSO) regression were first applied for variable screening, followed by multivariate logistic regression to pinpoint independent influencing factors. Random forest (RF), XGBoost, and deep learning models were constructed using Python, with performance evaluated using area under the curve (AUC). The optimal model was selected, and feature importance was analyzed.

**Results:**

Between the training and validation sets, no statistically significant baseline characteristic differences were found (*p *> 0.05). Multivariate logistic regression identified the apolipoprotein E epsilon 4 allele (*APOE *ε4) genotype, cerebrospinal fluid (CSF) p-tau181/amyloid-beta 42 (Aβ42) ratio, and diabetes as independent risk factors for AD (*p *< 0.05), while serum folate levels, Mini-Mental State Examination (MMSE) scores, and Montreal Cognitive Assessment (MoCA) scores served as independent protective factors (*p *< 0.05). In the validation set, the RF model achieved the highest AUC (0.879), followed by XGBoost (0.869) and deep learning (0.844), with the CSF p-tau181/Aβ42 ratio identified as the most predictive feature.

**Conclusion:**

The RF model, based on integrated multimodal clinical influencing factors and clinical indicators, demonstrates potential for AD risk stratification in high-risk populations when evaluated on a validation cohort.

## 1. Introduction

A progressive neurodegenerative disorder, Alzheimer’s disease (AD) is marked by cognitive dysfunction and memory decline. This condition severely diminishes patients’ quality of life and exacts a substantial toll on families and society [1,2]. Early diagnosis of AD remains challenging, as traditional diagnostic methods primarily rely on clinical symptoms and imaging examinations, which often lack sensitivity in the early stages [3]. With the recent progress in multimodal clinical influencing factors, information from multiple dimensions, such as genetics and biomarkers, has grown increasingly prominent in elucidating the pathogenesis of AD [4,5]. The apolipoprotein E epsilon 4 allele (*APOE *ε4) genotype has been confirmed as a major genetic risk factor for AD, while biomarkers such as the cerebrospinal fluid (CSF) p-tau181/amyloid-beta 42 (Aβ42) ratio and serum homocysteine levels are closely associated with AD progression [6,7]. Moreover, cognitive assessment tools like the Mini-Mental State Examination (MMSE) and Montreal Cognitive Assessment (MoCA) yield scores that reflect cognitive function and offer diagnostic utility [8]. However, single-dimensional indicators are insufficient for comprehensively unraveling the complex pathogenesis of AD and exhibit limited predictive efficacy. Machine learning algorithms, with their strong capabilities for data integration and pattern recognition, offer significant potential for improving disease risk prediction [9,10]. In parallel, noninvasive digital biomarkers such as handwriting analysis have shown promise for early AD detection by capturing subtle motor and cognitive impairments [11,12]. This study aimed to integrate multimodal clinical influencing factors and clinical indicators to construct a machine learning-based AD risk prediction model, thereby supporting risk stratification in high-risk populations and providing an adjunctive tool for early intervention and prevention efforts.

## 2. Materials and Methods

### 2.1 Study Participants

A total of 502 individuals at high risk of AD who visited our hospital were retrospectively included. Inclusion criteria: presence of cognitive decline (MMSE score <27 or MoCA score <26) or a family history of AD, age ≥60 years, and complete clinical data. Exclusion criteria: comorbid severe neurological disorders (e.g., Parkinson’s disease, cerebrovascular disease), severe cardiac, hepatic, or renal dysfunction, psychiatric disorders, and incomplete data or loss to follow-up. By simple randomization, participants were split in a 7:3 ratio into a training set of 350 and a validation set of 152. This study follows the STROBE guidelines (**Supplementary Material-STROBE-cohort_checklist**).

### 2.2 Data Collection

We extracted the following data from electronic health records and lab tests:

(1) Demographics: age, gender, education level, smoking history, alcohol consumption, and body mass index (BMI).

(2) Comorbidities: hypertension, diabetes, coronary heart disease, stroke, and dyslipidemia.

(3) Clinical scores: MMSE [13] and MoCA [14] scores.

(4) Biomarkers: CSF p-tau181/Aβ42 ratio, serum homocysteine, and serum folate.

(5) Genetic indicators: *APOE *ε4 genotype.

### 2.3 Outcome Definition

The primary outcome was the incidence of AD during follow-up. AD diagnosis was made by a panel of three neurologists based on the Chinese Guidelines for the Diagnosis and Treatment of AD (2021 Edition) [15], which incorporates clinical, neuropsychological, and biomarker evidence. Diagnosis was confirmed using clinical criteria combined with either cerebrospinal fluid (CSF) biomarkers (Aβ42, p-tau181) or amyloid positron emission tomography (PET) imaging when available. All diagnoses were made ante-mortem during follow-up. Patients were assessed at baseline and at 6‑month intervals during follow-up, with new diagnoses recorded at the time of first meeting diagnostic criteria. The median follow-up duration from enrollment to AD diagnosis or censoring was 2.4 years (interquartile range (IQR): 1.6–3.1 years).

### 2.4 Statistical Analysis

SPSS 26.0 (IBM Corp., Armonk, NY, USA) and R 4.2.3 (R Foundation for Statistical Computing, Vienna, Austria) were used for statistical analysis. Mean ± SD were used to describe normally distributed continuous variables, compared by *t*‑tests. Non‑normal continuous variables were presented as median (IQR) and analyzed with the Mann‑Whitney U test. Categorical variables were shown as counts (percentages) and compared using chi‑square tests. Univariate analysis was first run in the training set to detect factors associated with AD (*p *< 0.05). Significant variables were then subjected to least absolute shrinkage and selection operator (LASSO) regression for feature selection, with the optimal lambda.1se criterion applied to determine the final variables for multivariate logistic regression to identify independent predictors of AD. For machine learning prediction, random forest (RF) and XGBoost models were implemented using the selected variables as inputs to mitigate overfitting given the limited sample size, while still allowing the algorithms to assess feature importance internally. All machine learning models were implemented in Python 3.8.5 (Python Software Foundation, Beaverton, OR, USA) using scikit-learn (version 1.2.2, https://scikit-learn.org/). A deep learning model was also explored using TensorFlow (version 2.12.0, Google LLC, Mountain View, CA, USA) with Keras API, and its results are presented as a reference for exploratory comparison. Hyperparameter tuning was performed using 5‑fold cross‑validation with grid search. The final hyperparameters were: RF—n_estimators=200, max_depth=10, min_samples_split=5; XGBoost—learning_rate=0.1, max_depth=6, n_estimators=100, subsample=0.8; deep learning—a fully connected network with three hidden layers (64, 32, 16 neurons), ReLU activation, dropout rate 0.2, Adam optimizer, binary cross‑entropy loss, batch size 32, and 50 epochs with early stopping (patience=5). Receiver operating characteristic (ROC) curves were generated, and the area under the curve (AUC) was calculated to evaluate model performance. A calibration curve was plotted. Additionally, sensitivity, specificity, positive predictive value (PPV), negative predictive value (NPV), and Brier score (a calibration statistic measuring the mean squared difference between predicted probability and actual outcome) were computed to provide a comprehensive assessment of model performance. The model with the highest AUC was selected as the optimal model. Feature importance analysis was conducted using the Gini impurity index in the training set to calculate predictive contribution, with features sorted in descending order. Decision curve analysis (DCA) was used to evaluate the clinical application value of the nomogram by calculating the net benefit at different threshold probabilities. A *p* value < 0.05 was considered statistically significant.

## 3. Results

### 3.1 Comparison of Baseline Characteristics Between Training and Validation Sets

Among the 350 patients in the training set, 98 (28.00%) developed AD, while 252 (72.00%) did not. In the validation set (n = 152), 42 (27.63%) developed AD and 110 (72.37%) did not. Baseline features were not significantly different between the two cohorts (*p *> 0.05) (Table 1).

**Table 1.** **Comparison of baseline characteristics between training and validation sets**.

Variable	Training set (n = 350)	Validation set (n = 152)	*t/χ^2^*	*p*
Age (years)	72.25 ± 6.67	73.55 ± 8.51	1.839	0.066
Gender (male/female (%))	196/154 (56.00/44.00)	82/70 (53.95/46.05)	0.181	0.671
Education level (years)	8.66 ± 3.19	8.52 ± 3.52	0.594	0.553
Smoking history (yes/no)	88/262 (25.14/74.86)	40/112 (26.32/73.68)	0.077	0.782
Alcohol consumption (yes/no)	65/285 (18.57/81.43)	30/122 (19.74/80.26)	0.094	0.759
Hypertension (yes/no)	154/196 (44.00/56.00)	60/92 (39.47/60.53)	0.888	0.346
Diabetes (yes/no)	70/280 (20.00/80.00)	32/120 (21.05/78.95)	0.073	0.788
Coronary heart disease (yes/no)	68/282 (19.43/80.57)	32/120 (21.05/78.95)	0.175	0.676
*APOE *ε4 genotype (carrier/non-carrier)	121/229 (34.57/65.43)	50/102 (32.89/67.11)	0.133	0.716
CSF p-tau181/Aβ42 ratio	0.27 ± 0.11	0.29 ± 0.13	1.769	0.078
Serum homocysteine (μmol/L)	14.85 ± 4.34	15.32 ± 4.65	1.091	0.276
Serum folate (nmol/L)	12.51 ± 3.55	13.12 ± 3.15	1.829	0.068
MMSE score	24.61 ± 3.37	24.89 ± 3.52	0.844	0.399
MoCA score	22.78 ± 3.51	23.21 ± 3.52	1.261	0.208
BMI (kg/m^2^)	24.47 ± 3.15	25.02 ± 3.12	1.803	0.072
Dyslipidemia (yes/no)	109/241 (31.14/68.86)	45/107 (29.61/70.39)	0.118	0.731
Stroke history (yes/no)	35/315 (10.00/90.00)	16/136 (10.53/89.47)	0.032	0.858

*APOE *ε4, apolipoprotein E epsilon 4 allele; CSF, cerebrospinal fluid; Aβ42, amyloid-beta 42; MMSE, Mini-Mental State Examination; MoCA, Montreal Cognitive Assessment; BMI, body mass index.

### 3.2 Univariate Analysis of Factors Influencing AD Onset

In training set, univariate analysis showed that statistically significant differences (*p *< 0.05) were observed between the early-onset and non-onset groups in the training set for the following indicators: diabetes mellitus, coronary heart disease, *APOE *ε4 genotype, CSF p-tau181/Aβ42 ratio, serum homocysteine, serum folate, MMSE score, and MoCA score (Table 2).

**Table 2.** **The result of univariate analysis of factors influencing AD onset in training set**.

Variable	Onset group (n = 98)	Non-onset group (n = 252)	*t/χ^2^*	*p*
Age (years)	73.25 ± 7.01	71.86 ± 6.52	1.753	0.081
Gender (male/female (%))	54/44 (55.11/44.89)	142/110 (56.35/43.65)	0.045	0.833
Education level (years)	8.26 ± 3.28	8.82 ± 3.15	1.476	0.141
Smoking history (yes/no)	26/72 (26.53/73.47)	62/190 (24.61/75.39)	0.139	0.709
Alcohol consumption (yes/no)	17/81 (17.35/82.65)	48/204 (19.05/80.95)	0.135	0.713
Hypertension (yes/no)	42/56 (42.86/57.14)	112/140 (44.44/55.56)	0.072	0.788
Diabetes (yes/no)	28/70 (28.57/71.43)	42/210 (16.67/83.33)	6.327	0.012
Coronary heart disease (yes/no)	28/70 (28.57/71.43)	40/212 (15.87/84.13)	7.268	0.007
*APOE *ε4 genotype (carrier/non-carrier)	58/40 (59.18/40.82)	63/189 (25.00/75.00)	36.451	0.001
CSF p-tau181/Aβ42 ratio	0.38 ± 0.12	0.23 ± 0.08	13.563	0.001
Serum homocysteine (μmol/L)	16.35 ± 4.57	14.28 ± 4.12	4.091	0.001
Serum folate (nmol/L)	10.58 ± 3.15	13.25 ± 3.42	6.701	0.001
MMSE score	21.85 ± 3.02	25.68 ± 2.85	11.101	0.001
MoCA score	20.86 ± 3.41	23.52 ± 3.26	6.766	0.001
BMI (kg/m^2^)	24.78 ± 3.25	24.35 ± 3.12	1.144	0.253
Dyslipidemia (yes/no)	31/67 (31.63/68.37)	78/174 (30.95/69.05)	0.015	0.902
Stroke history (yes/no)	10/88 (10.21/89.79)	25/227 (9.92/90.08)	0.006	0.937

### 3.3 Multivariable Logistic Regression Analysis of Factors Influencing AD Onset

The occurrence of AD was used as the dependent variable (Non-AD group = 0, AD = 1). LASSO regression included the statistically significant variables from univariate analysis for variable selection, with optimal variables chosen via the lambda.1se criterion (**Supplementary Figs. 1,2**). The most suitable predictors included diabetes, *APOE *ε4 genotype, CSF p-tau181/Aβ42 ratio, serum folate level, MMSE score, and MoCA score. These variables were subsequently incorporated into the multivariable logistic regression analysis. The results demonstrated that diabetes (OR = 6.212, 95% CI: 1.829–21.112, *p *= 0.003), *APOE *ε4 genotype (OR = 5.196, 95% CI: 2.517–9.934, *p *= 0.001), and CSF p-tau181/Aβ42 ratio (OR = 2.051, 95% CI: 1.358–3.094, *p *= 0.001) were independent risk factors for AD onset. Conversely, serum folate level (OR = 0.814, 95% CI: 0.722–0.917, *p *= 0.001), MMSE score (OR = 0.633, 95% CI: 0.542–0.740, *p *= 0.001), and MoCA score (OR = 0.813, 95% CI: 0.724–0.912, *p *= 0.001) were identified as independent protective factors (Table 3).

**Table 3.** **The result of multivariable logistic regression analysis**.

Variable	β	SE	Wald	*p*	OR	95% CI
Diabetes	1.827	0.624	8.571	0.003	6.212	1.829–21.112
*APOE *ε4 genotype	1.609	0.350	21.140	0.001	5.196	2.517–9.934
CSF p-tau181/Aβ42 ratio	0.718	0.210	11.690	0.001	2.051	1.358–3.094
Serum folate	–0.206	0.061	11.428	0.001	0.814	0.722–0.917
MMSE score	–0.457	0.080	32.774	0.001	0.633	0.542–0.740
MoCA score	–0.208	0.059	12.443	0.001	0.813	0.724–0.912

### 3.4 Predictive Performance of Machine Learning Models in Training and Validation Sets

**Fig. 1.**
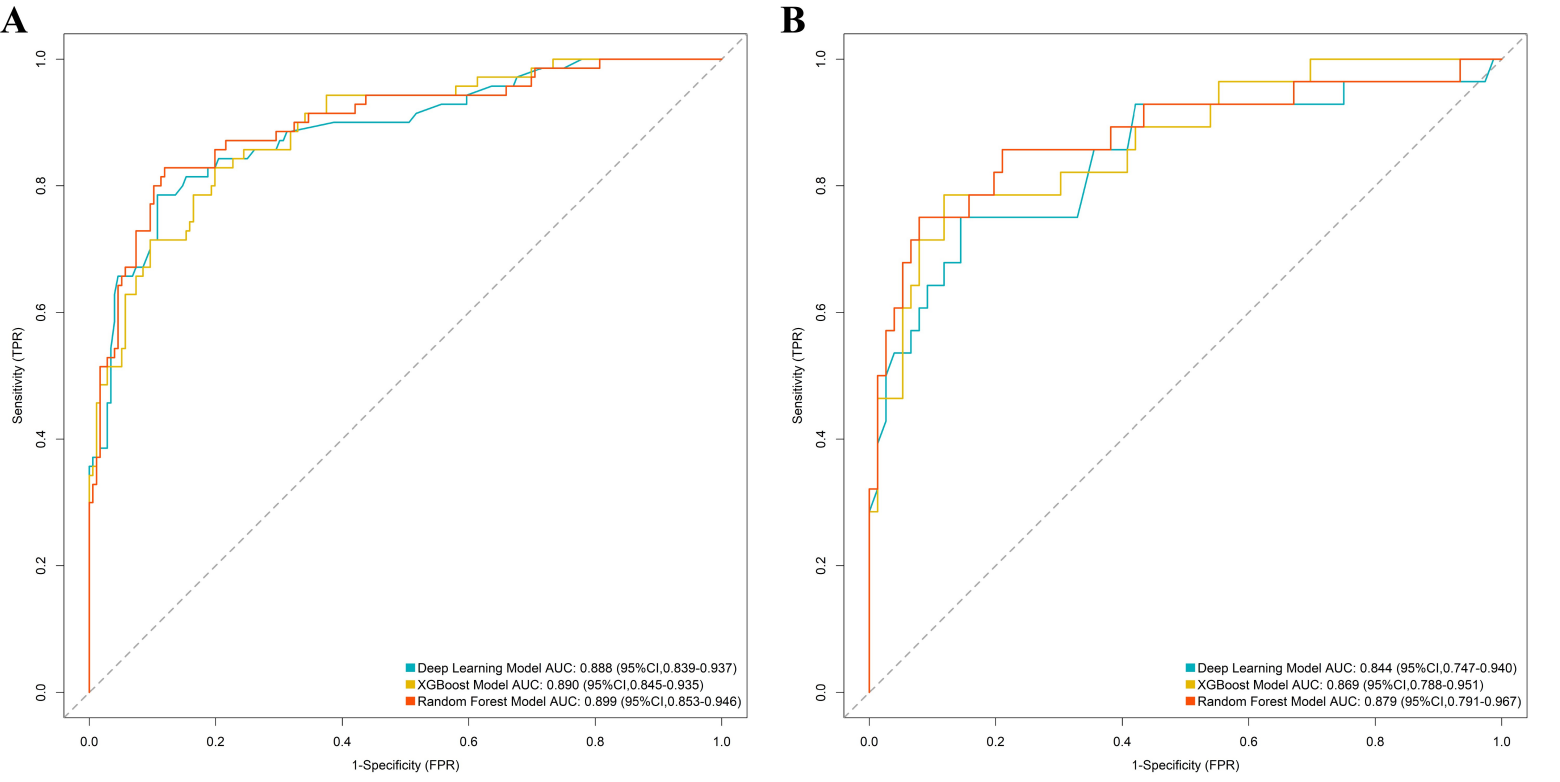
**Receiver operating characteristic curves of machine learning models (A: Training set; B: Validation set)**. Note: AUC, Area under the curve. AUC values are shown for random rorest, XGBoost, and deep learning models. The RF model achieved the highest AUC in both sets (0.899 in training, 0.879 in validation). TPR, true positive rate; FPR, false positive rate.

**Fig. 2.**
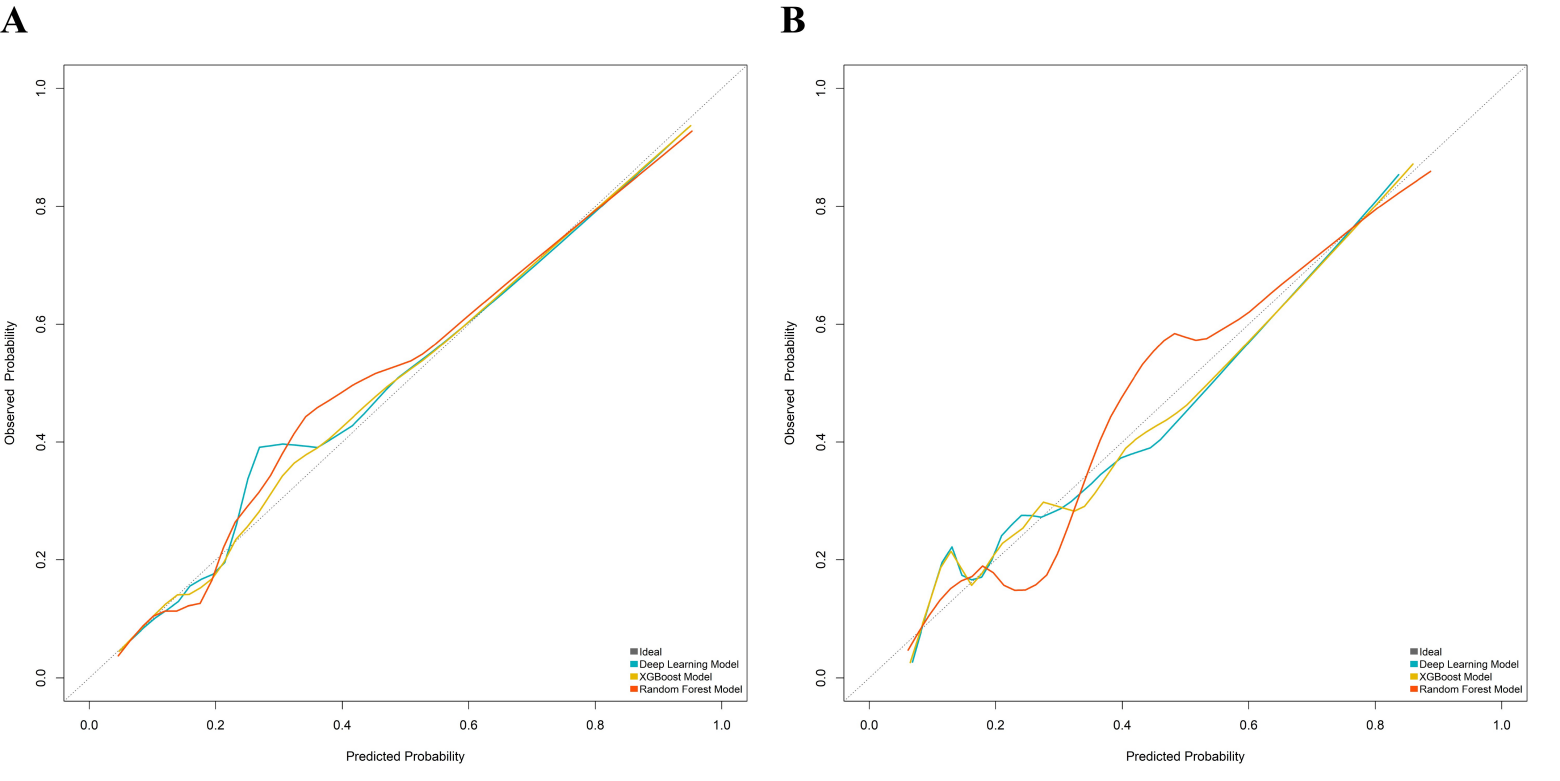
**Calibration curves of machine learning models (A: Training set; B: Validation set)**. Note: The diagonal dashed line represents perfect calibration, where predicted probabilities equal observed outcomes. Closer proximity of the model curves to the diagonal line indicates better agreement between predicted and actual Alzheimer’s disease (AD) onset probabilities. The random forest model demonstrated good calibration in the training set and slight systematic overestimation in the 0.3–0.5 risk range.

The RF, XGBoost, and deep learning models were evaluated in both training and validation sets, with their predictive performance summarized in Table 4. In the training set, RF achieved an AUC of 0.899, XGBoost of 0.890, and deep learning of 0.888. In the validation set, RF achieved an AUC of 0.879, XGBoost of 0.869, and deep learning of 0.844. Regarding sensitivity and specificity, RF performed slightly better (0.835 and 0.828, respectively) compared to XGBoost (0.822 and 0.820) and deep learning (0.815 and 0.814). The RF model exhibited the highest AUC in both sets (Fig. 1). In the validation set, the calibration curves showed that XGBoost and deep learning remained close to the ideal diagonal line, while RF exhibited systematic miscalibration in the 0.3–0.5 risk range, overestimating risk compared to observed probabilities (Fig. 2).

**Table 4.** **Predictive performance of machine learning models in the training and validation sets**.

Model	Dataset	AUC	Sensitivity	Specificity	PPV	NPV	Brier score
Random forest	Training	0.899 (0.853–0.946)	0.842	0.832	0.641	0.934	0.156
	Validation	0.879 (0.791–0.967)	0.835	0.828	0.635	0.931	0.163
XGBoost	Training	0.890 (0.845–0.935)	0.829	0.827	0.635	0.931	0.134
	Validation	0.869 (0.788–0.951)	0.822	0.820	0.630	0.929	0.141
Deep learning	Training	0.888 (0.839–0.937)	0.821	0.819	0.628	0.928	0.148
	Validation	0.844 (0.747–0.940)	0.815	0.814	0.620	0.926	0.155

AUC, area under the curve; PPV, positive predictive value; NPV, negative predictive value.

Although RF achieved the highest AUC (0.899), its calibration was inferior to XGBoost. Given the comparable discrimination (AUC difference 0.009, DeLong’s test *p* = 0.23) and better calibration of XGBoost, we consider both models as viable options, with XGBoost potentially more suitable for clinical applications requiring well-calibrated risk estimates.

### 3.5 Construction of the AD Risk Prediction Model

**Fig. 3.**
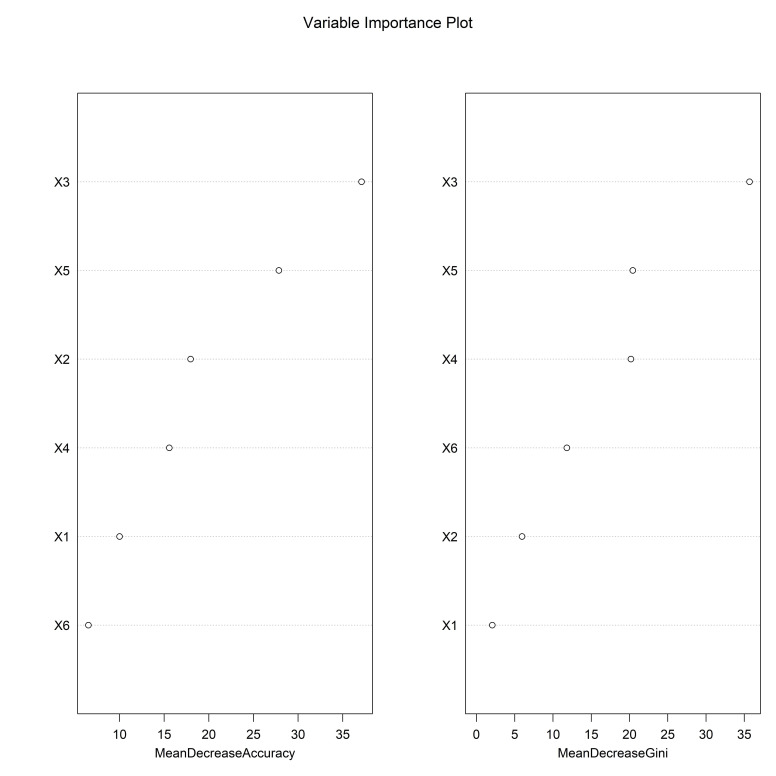
**Feature importance ranking in the random forest model**. Note: X1: Diabetes; X2: *APOE *ε4 genotype; X3: CSF p-tau181/Aβ42 ratio; X4: Serum folate; X5: MMSE score; X6: MoCA score. The importance scores were derived from the mean decrease Gini across all decision trees in the random forest model. The horizontal axis represents the relative importance score of each predictor, and the vertical axis lists the top six variables selected by the model.

Feature importance analysis based on the RF model ranked CSF p-tau181/Aβ42 ratio, MMSE score, *APOE *ε4 genotype, serum folate level, diabetes, and MoCA score as the most critical predictive factors (**Supplementary Fig. 3 **and Fig. 3).

### 3.6 Decision Curve Analysis

**Fig. 4.**
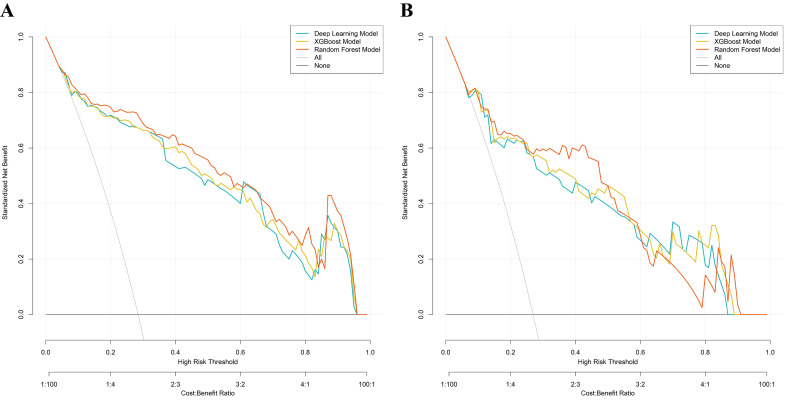
**Decision curves of the machine learning model (A: Training set; B: Validation set)**. Note: The y-axis represents the net benefit, and the x-axis represents the threshold probability. The gray horizontal line indicates the assumption that no patients have AD (treat none), while the gray slanted line indicates the assumption that all patients have AD (treat all).

The DCA showed that the proposed model provided higher net benefit across threshold probabilities between 0.10 and 0.80, suggesting clinical utility for guiding early intervention. The choice of threshold may depend on the balance between the harms of intervention (e.g., patient anxiety, healthcare resource use) and the benefit of early detection, which should be determined in shared decision‑making with patients (Fig. 4).

### 3.7 Sensitivity Analysis

To evaluate the model’s potential for early screening without relying on cognitive scores, we re-ran the RF model excluding MMSE and MoCA. The model retained all other variables (demographics, medical history, *APOE *ε4, CSF p‑tau181/Aβ42 ratio, serum homocysteine, and serum folate). In the validation set, the AUC decreased to 0.823 (95% CI: 0.786–0.860), with a sensitivity of 0.782 and a specificity of 0.803. Feature importance shifted, with CSF p‑tau181/Aβ42 ratio and *APOE *ε4 genotype becoming the top predictors. These results suggested that while cognitive scores enhance predictive accuracy, the model retains moderate discriminatory ability using only biomarkers and clinical history, supporting its potential for earlier risk stratification.

## 4. Discussion

The high disability rate and subtle onset of AD—the globe’s most common neurodegenerative disorder—create serious challenges for public health systems [16]. Currently, early diagnosis of AD primarily relies on clinical symptoms and neuroimaging, yet these methods exhibit limited sensitivity during the prodromal stage, hindering precise intervention. Recent advances in multimodal clinical influencing factors technologies have provided new insights into the complex pathological mechanisms of AD, while machine learning algorithms offer the potential to overcome limitations of traditional prediction models by integrating multidimensional data [17,18,19].

This study retrospectively analyzed clinical data from 502 high-risk AD individuals and developed a risk prediction model based on multimodal clinical influencing factors and clinical indicators. According to the results from the validation set, the RF model performed best (AUC = 0.879), with key predictors including the CSF p‑tau181/Aβ42 ratio, *APOE* ε4 genotype, and MMSE scores. These findings confirm that machine learning models integrating genetic, biomarker, and clinical data significantly enhance AD risk prediction accuracy, providing a reliable tool for early screening and personalized prevention.

To overcome the limitations of single-dimensional AD research, it is vital to integrate multimodal clinical influencing factors data (genetic, biomarker, and clinical indicators). AD pathogenesis involves genetic susceptibility, abnormal protein deposition, metabolic dysregulation, and cognitive decline, yet individual markers (e.g., *APOE* ε4 genotype or CSF biomarkers) only reflect isolated pathological changes, failing to capture the comprehensive disease progression [20,21]. This study incorporated multi-dimensional indicators, including *APOE* ε4 genotype (genomics), CSF p-tau181/Aβ42 ratio (proteomics), serum folate (metabolomics), and MMSE scores (clinical phenotype). After reducing redundant variables using LASSO regression, machine learning algorithms were employed to construct predictive models, with the RF model exhibiting superior performance in the validation set. This highlights the advantage of multimodal clinical influencing factors integration—while traditional logistic regression captures only linear relationships, RF, through ensemble decision trees, effectively handles feature interactions (e.g., synergistic effects between *APOE* ε4 and CSF biomarkers) and mitigates noise in high-dimensional data.

The incorporation of electronic health records (EHRs) further enhanced the model’s clinical utility. This study systematically collected demographic characteristics, medical history, and laboratory test results from EHRs, enabling standardized integration of multi-source data and avoiding fragmentation issues inherent in traditional data collection [22]. Machine learning algorithms identified several important predictive factors, including established risk factors (e.g., diabetes) and biomarkers (e.g., serum folate, cognitive scores). Feature importance analysis ranked these variables based on their individual contribution to predictions, but further analyses such as SHapley Additive exPlanations interaction plots or partial dependence plots are needed to explore potential interactions between variables. Compared to XGBoost (validation AUC = 0.869) and deep learning (validation AUC = 0.844), RF demonstrated superior performance in the validation set, possibly due to its stronger ability to capture nonlinear relationships and robustness against outliers, making it more suitable for clinical datasets with skewed distributions and missing values. Beyond the multimodal clinical data used in this study, handwriting‑based machine learning approaches have also demonstrated high accuracy in AD prediction, offering a non‑invasive and scalable alternative for risk screening [23,24]. It should be noted that the deep learning model was included as an exploratory comparison; given the low feature dimensionality and moderate sample size, its performance did not surpass that of the tree‑based ensemble models, which is consistent with expectations.

DCA further evaluated the clinical utility of the proposed model. The results showed that the model provided a higher net benefit than the “treat all” and “treat none” strategies across threshold probabilities ranging from 0.10 to 0.80, indicating its clinical value across different risk preferences. In the clinical context of this study, patients identified as high-risk by the model could be considered for more intensive follow-up (e.g., semi-annual cognitive assessments) and early lifestyle or medical interventions (e.g., cognitive training, glucose and blood pressure management). The choice of risk threshold should balance the potential harms of intervention (such as patient anxiety and overuse of healthcare resources) against the benefits of early detection. The appropriate threshold should be determined through shared decision-making between clinicians and patients based on individual circumstances.

Multivariate logistic regression identified six independent risk factors, elucidating key mechanisms of AD pathogenesis across genetic, pathophysiological, and clinical dimensions. The *APOE* ε4 genotype, the most established genetic risk factor for AD, exhibited the strongest association (OR = 5.196, *p* < 0.001), consistent with prior findings that *APOE* ε4 accelerates neurodegeneration by promoting Aβ deposition and tau hyperphosphorylation [25,26]. *APOE* ε4 carriers in this study had over 10 times higher AD risk than non-carriers, ranking third in feature importance in the RF model, underscoring its irreplaceable predictive value in multimodal clinical influencing factors models [27]. The CSF p-tau181/Aβ42 ratio showed the highest predictive importance (OR = 2.051, *p *< 0.001), reflecting AD’s hallmark pathological changes—impaired Aβ clearance and tau aggregation. Elevated ratios may precede clinical symptoms by years, suggesting biomarkers’ central role in early detection.

Diabetes, a modifiable risk factor (OR = 3.161, *p *= 0.003), may contribute to AD through insulin resistance-induced cerebrovascular damage, oxidative stress, and impaired Aβ clearance [28]. These findings support integrating diabetes management into AD prevention strategies [29,30]. Serum folate levels emerged as a protective factor (OR = 0.814, *p *< 0.001), possibly mediated by homocysteine metabolism—folate deficiency elevates homocysteine, exacerbating cognitive decline through endothelial damage and amyloid deposition. Folate supplementation may serve as a low-cost preventive measure, though optimal dosing requires further validation [31]. MMSE (OR = 0.633, *p *< 0.001) and MoCA scores (OR = 0.813, *p *< 0.001) as cognitive assessment tools demonstrated protective effects, directly linking clinical phenotypes to pathological progression [32,33]. MMSE ranked second in feature importance in this RF model, while MoCA also contributed to the predictions. Both cognitive assessments provide complementary information, and their rankings reflect their respective roles in this specific predictive context.

Several limitations should be acknowledged. First, the inclusion of MMSE and MoCA scores may introduce circularity bias, as cognitive scores were both a key component of the inclusion criteria and overlapped with outcome definition. This may overestimate the model’s performance in true screening settings. Accordingly, the model developed in this study is better positioned as an adjunct tool for risk stratification in high-risk populations rather than a replacement for existing clinical screening approaches. Second, the single‑center retrospective design lacks external validation and may introduce regional sample bias, necessitating future multi‑center studies. Third, the moderate sample size may limit the detection of rare variants; expanding the cohort could enhance robustness. Fourth, neuroimaging markers (e.g., hippocampal volume, amyloid PET) were not included due to incomplete electronic health record data, although their inclusion could further optimize predictions. Additionally, the study lacked longitudinal data to evaluate the temporal evolution of individual risk, which is critical for dynamic prediction and personalized intervention timing.

## 5. Conclusion

This study showed that machine learning models integrating multimodal clinical data achieved good discrimination for AD risk prediction in our validation cohort. Although RF achieved the numerically highest AUC in the validation set, XGBoost demonstrated better calibration, highlighting the importance of considering both discrimination and calibration in model selection. Future research should validate model generalizability through multi-center external validation studies and explore integration strategies incorporating neuroimaging to advance precision prevention frameworks for AD.

## Data Availability

The data that support the findings of this study are available from the corresponding author upon reasonable request. For full reproducibility, the analysis code and model implementation have been deposited in a public GitHub repository and can be accessed at: (https://github.com/lovecoldwater/AD-Risk-Prediction).
