# Supplementary material for: AI-Enabled Modeling for Alzheimer’s Disease Risk Prediction and Validation
Source: Rev Neurol. 2026 Jul 21;81(7):49220. doi: 10.31083/RN49220 (PMC13421099; doi:10.31083/RN49220)
Supplement: Supplementary file 1 [file 1576-6578-81-7-49220-s1.zip › Supplementary Material.docx]

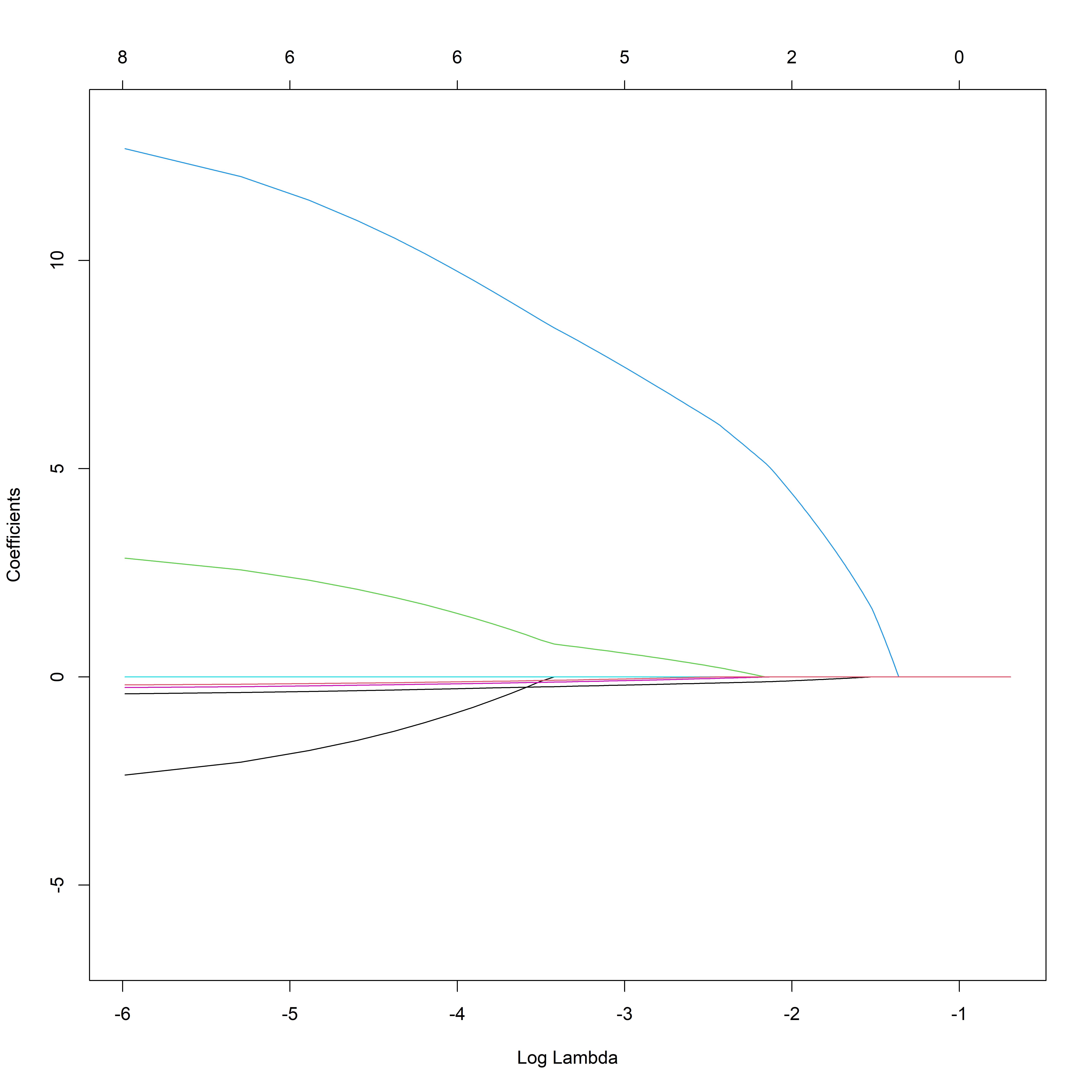


**Supplementary Fig. 1.** LASSO regression analysis plot


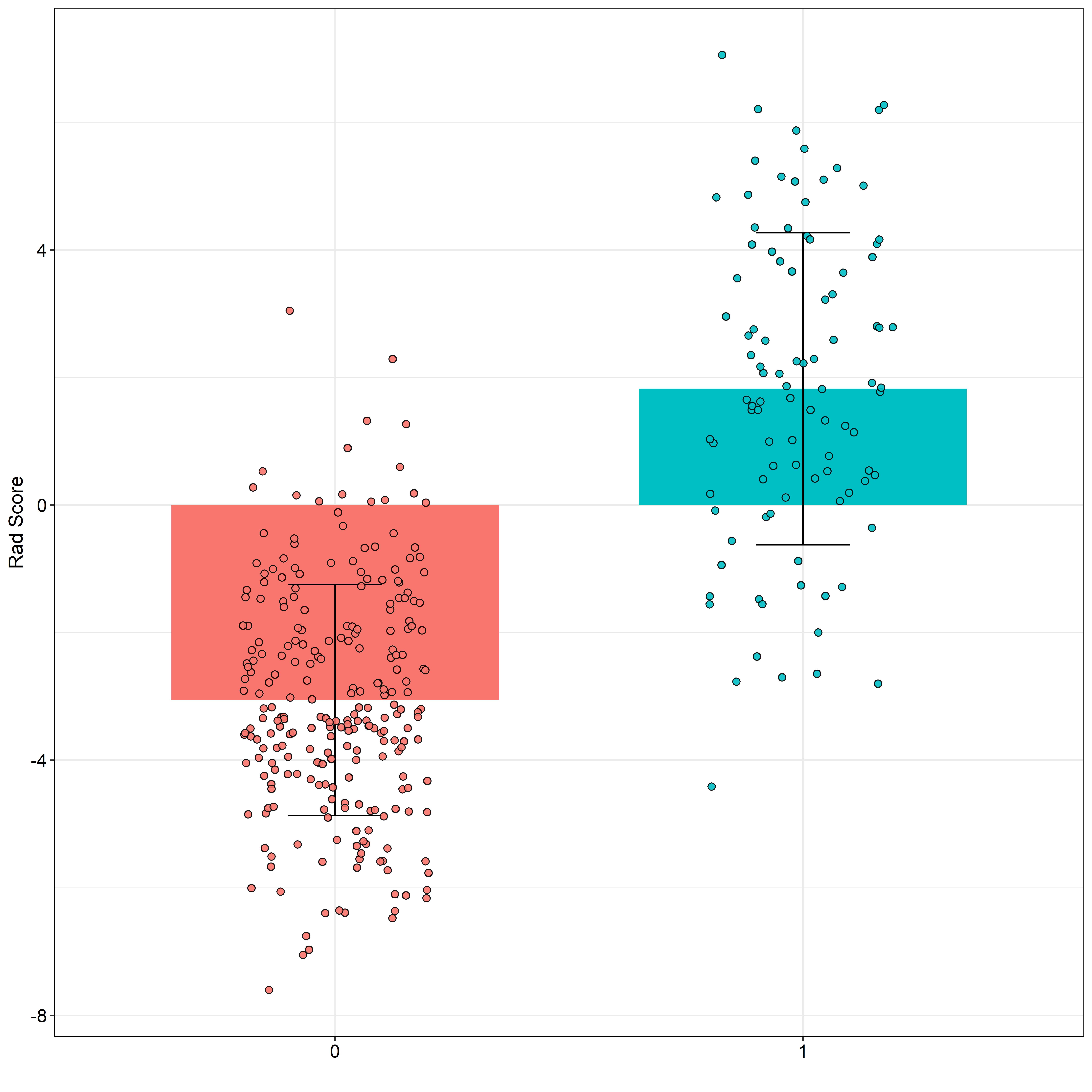


**Supplementary Fig. 2.** Comparison of LASSO score differences


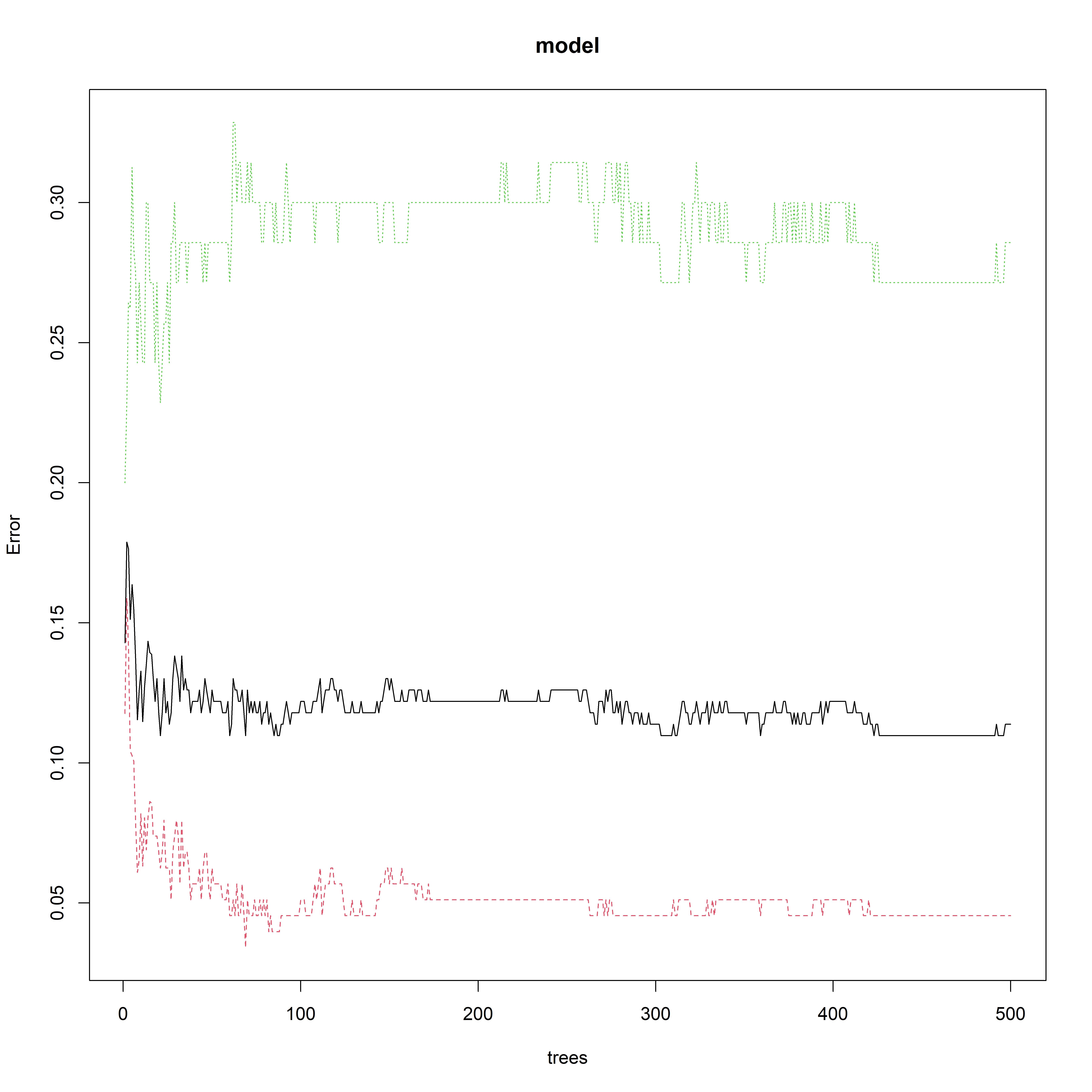


**Supplementary Fig. 3.** Trend of out-of-bag error rate with increasing decision tree numbers in the random forest model.

Note: The out-of-bag error stabilizes after approximately 100 trees, indicating model convergence.
